# Relationship between Joint Position Sense, Force Sense, and Muscle Strength and the Impact of Gymnastic Training on Proprioception

**DOI:** 10.1155/2018/5353242

**Published:** 2018-02-18

**Authors:** Bartłomiej Niespodziński, Andrzej Kochanowicz, Jan Mieszkowski, Elżbieta Piskorska, Małgorzata Żychowska

**Affiliations:** ^1^Department of Anatomy and Biomechanics, Institute of Physical Education, Kazimierz Wielki University in Bydgoszcz, Sportowa 2, 85-091 Bydgoszcz, Poland; ^2^Department of Gymnastics and Dance, Gdansk University of Physical Education and Sport, Kazimierza Górskiego 1, 80-336 Gdańsk, Poland; ^3^Department of Pathobiochemistry and Clinical Chemistry, Nicolaus Copernicus University Collegium Medicum, M. Curie Skłodowskiej 9, 85-094 Bydgoszcz, Poland; ^4^Department of Life Sciences, Faculty of Physical Education, Gdansk University of Physical Education and Sport, Kazimierza Górskiego 1, 80-336 Gdańsk, Poland

## Abstract

The aims of this study were (1) to assess the relationship between joint position (JPS) and force sense (FS) and muscle strength (MS) and (2) to evaluate the impact of long-term gymnastic training on particular proprioception aspects and their correlations. 17 elite adult gymnasts and 24 untrained, matched controls performed an active reproduction (AR) and passive reproduction (PR) task and a force reproduction (FR) task at the elbow joint. Intergroup differences and the relationship between JPS, FS, and MS were evaluated. While there was no difference in AR or PR between groups, absolute error in the control group was higher during the PR task (7.15 ± 2.72°) than during the AR task (3.1 ± 1.93°). Mean relative error in the control group was 61% higher in the elbow extensors than in the elbow flexors during 50% FR, while the gymnast group had similar results in both reciprocal muscles. There was no linear correlation between JPS and FS in either group; however, FR was negatively correlated with antagonist MS. In conclusion, this study found no evidence for a relationship between the accuracy of FS and JPS at the elbow joint. Long-term gymnastic training improves the JPS and FS of the elbow extensors.

## 1. Introduction

Muscle strength (MS) is one of the most important factors affecting human performance. It allows athletes to overcome external load applied to the body and thus allows movement. While many studies have investigated ontogenetic [1, 2] and training-induced [3, 4] development of MS, little attention has been paid to the proprioceptive system which controls force production. According to Proske and Gandevia [5], proprioception is the sense of relative localization and movement of the body in space and the sense of tension, effort, and balance. The nervous system receives information from proprioceptors located in muscles (muscle spindles), tendons (Golgi tendon organs), joints, and the skin, which transmit afferent information regarding mechanical stimuli generated within the musculoskeletal system [6]. The afferent information reaches the central nervous system, where it is processed along with the corollary induced information of the effort to control the body position and movement and the sense of force [5].

Studies investigating proprioception have focused mainly on joint position sense (JPS) or kinesthesia, while interest in force sense (FS) is limited. FS is possible due to the nervous system integrating tensile information from muscular proprioceptors (muscle spindles and Golgi tendon organs) with the sense of effort induced centrally [7, 8]. At present, researchers do not agree as to which of the above mechanisms plays the dominant role in FS [9].

There is some limited evidence for an association between MS and proprioception. Several studies have shown that greater MS is associated with improved balance control [10–12] and strength training has been found to improve JPS at the shoulder [13]. Authors also point out that accurate proprioception at the shoulder joint is possible due to balanced strength between reciprocal muscles [14]. As muscle spindles are the primary proprioceptors involved in JPS [15] and are also involved in FS [16–18], there may be a relationship between these two aspects of proprioception and MS.

Research regarding the relationship between FS and JPS is also limited. Kim et al. [19] found no correlation between JPS and FS at the ankle joint in subjects with functional ankle instability and in an uninjured control group; however, authors acknowledge that participants varied in physical activity, gender, and level of injury. Moreover, as FS is more accurate in the upper limb [20], research at the elbow joint may yield different results. A good proprioception in the elbow joint is necessary to achieve high performance in sports like gymnastics, baseball, and basketball and many others [21, 22]. On the other hand, sport activities often lead to injuries which impair proprioception in the elbow joint. Therefore, the first aim of this study was to establish the relationship between FS, JPS, and MS at the elbow joint. While strength training interferes with JPS [13, 23], the authors hypothesized that FS would be positively correlated with MS.

Previous research has found that sport and strength training can affect JPS [13, 24, 25], while research regarding FS is limited. Therefore, the second aim of this study was to compare the accuracy of FS and JPS in athletes and nonathletes using gymnasts as the athlete group as gymnastics requires both excellent upper body strength and excellent precision in muscle tension. It was hypothesized that the gymnast group would demonstrate greater accuracy in JPS and FS in comparison to untrained adults. Despite the overall impact of gymnastics on upper body strength, training is directed at developing extensor strength in particular [26]. The impact of training on FS in flexors and extensors may therefore be different.

## 2. Materials and Methods

### 2.1. Participants

Seventeen elite adult gymnasts and 24 age-matched untrained controls were recruited. Their basic anthropometrical characteristics are shown in Table 1. All athletes were in the competitive training phase and trained for about 24 hours in six training sessions per week. The control group was comprised of physically active Tourism and Recreation students, none of whom participated in structured sports training. The criterion for participants' inclusion was no medical history of injuries or neuromuscular disorders that may have affected the elbow joint. Additionally, athletes had to practice artistic gymnastics since they were six or seven years old and compete at least at a national level. The study was approved by the Bioethical Commission and all participants signed informed consent forms.

### 2.2. Procedures

This study consisted of two parts. In part one, body composition analysis and muscle strength evaluation were conducted. In part two, JPS and FS at the elbow joint were evaluated. Both parts took place in the morning about 2-3 hours after the first meal and in a state of good hydration. Participants were prohibited from exercising 24 hours prior to the study. In the case of the gymnasts, measurements were performed on Mondays before the first training due to the fact that Sunday was the only day of rest for gymnasts.

Body composition analysis was conducted using the InBody 770 bioelectrical impedance analyzer (BioSpace, Korea) in the morning prior to breakfast. Body fat and lean arm mass were recorded and archived for torque normalization purposes. Prior to strength testing, participants completed a warm-up on the Monark 891E hand cycle ergometer (Monark Exercise AB, Sweden) involving five minutes of arm cranking using a power output of 1 W/kg and a crank rate of 60 rev/min. Maximal MS at the elbow was then tested using the Biodex System 4 isokinetic dynamometer (Biodex Medical Systems, Inc., Shirley, NY, USA). Each participant sat with his/her dominant elbow resting on a leather support. The position of each participant was adjusted to obtain 45° and 90° angles at the glenohumeral and elbow joints, respectively. Participants' position was the same for all procedures. Limb dominance was determined by participants' preferred writing hand. Participants performed three isometric 4 s maximal voluntary contractions (MVC) for elbow flexion and extension in random order. Between each repetition, participants had at least 30 s rest to reduce fatigue. During testing, participants had visual feedback and were given verbal encouragement. The highest peak torque from three trials was taken for analysis.

JPS was then evaluated using the Biodex System 4 isokinetic dynamometer. The reliability of this device in JPS assessment of the elbow and other joints was previously shown [27, 28]. Prior to testing, participants were familiarized with the test using different random target angles. During testing, participants made three attempts to reproduce (remember) a 90° angle at the elbow joint without visual cues both actively (AR) and passively (PR) using their dominant limb. Before each attempt to reproduce the target angle, participants' elbow joints were set to the target angle from a random starting point, and they were asked to feel and remember the position. Participants began in a neutral position at the elbow (full extension, 0°) where an MVC of the elbow flexors was performed to condition the muscle and induce thixotropic effect during PR [29]. Participants were then asked to reproduce (always in the direction of flexion) the target angle beginning from a random start position and indicate that they reached it by pressing a button held in the contralateral hand. During PR testing, the participant's elbow was moved passively by the device at a constant motion of 0.5°·s^−1^. During AR testing, participants' actively flexed their elbow, stopping at the point where they felt they had reached 90° flexion. Different reproduction modes were used to evaluate whether muscle thixotropy would affect the analyzed relationships. To assess the accuracy and direction of bias of the JPS performance, the absolute and constant errors were calculated, respectively. The constant error was calculated as the mean of difference between the target angle and the recorded reproduction angle in three trials. The absolute error was calculated as the previous one, but the absolute difference between the target and the reproduced angle was taken to analysis.

FS was then evaluated using the Biodex System 4 isokinetic dynamometer using the unilateral remembered force reproduction (FR) test. The high reliability of FR measurements using the device was shown previously [30]. It should be noted that this test assesses torque reproduction rather than force, but for convenience, it will be described as FR. The target force was displayed via visual feedback to participants given the opportunity to familiarize themselves with the task. The participants were blindfolded and asked to reproduce the target force and sustain it for 4 s. Participants were tested at 20% and 50% of MVC at elbow flexors and extensors in random order. A one-minute rest was given between trials to avoid fatigue. The mean relative error (mean absolute difference between target and reproduction torque during FR) for accuracy and the range of relative error for force steadiness expressed in % MVC were recorded. To evaluate the direction of bias, the constant error was also calculated as the mean difference between the target and the reproduction torque during FR (% MVC). Three trials were conducted at each force level with the mean taken for analysis.

### 2.3. Statistical Analysis

Intergroup comparison of anthropometric and strength characteristics was assessed using unpaired *t*-tests. Pearson's correlation coefficient was calculated to study the relationship between FS, JPS, and MS. It was performed separately for each group to assess the impact of long-term gymnastic training on these relations. The magnitude of the effect size of the correlation was evaluated according to Cohen [31] with its further modification by Hopkins [32].

Two sets of ANOVA tests were then performed. First, two-way (2* groups* × 2* muscles*) ANOVAs of repeated measures were performed to compare FS performance between gymnasts and the controls in two reciprocal muscle groups (elbow flexors and extensors). Second, two-way (2* groups* × 2* modes*) ANOVAs of repeated measures were performed to compare JPS performance between two groups in AR and PR. Shapiro-Wilk and Levene's tests were performed to check the normal distribution and the homogeneity of variance, respectively. The level of significance was set at *α* = 0.05 for all the tests. All analyses were performed with Statistica 12 (StatSoft Inc., Tulsa, OK, USA). To assess the required sample size, the power analysis for interactions between effects in two-way ANOVA of repeated measures was conducted using G*∗*Power ver. 3.1.9.2 software [33]. It was shown that the minimal total sample size for the medium effect size *f* with power of 0.8 and 0.05 level of significance was equal to 24 subjects.

## 3. Results

### 3.1. Muscle Strength

Results for isometric peak torque produced by elbow flexors and extensors are shown in Table 1. Elbow flexion peak torque was greater in the gymnast group, while no difference was found for elbow extension between groups.

### 3.2. Joint Position Sense

**Figure 1:**
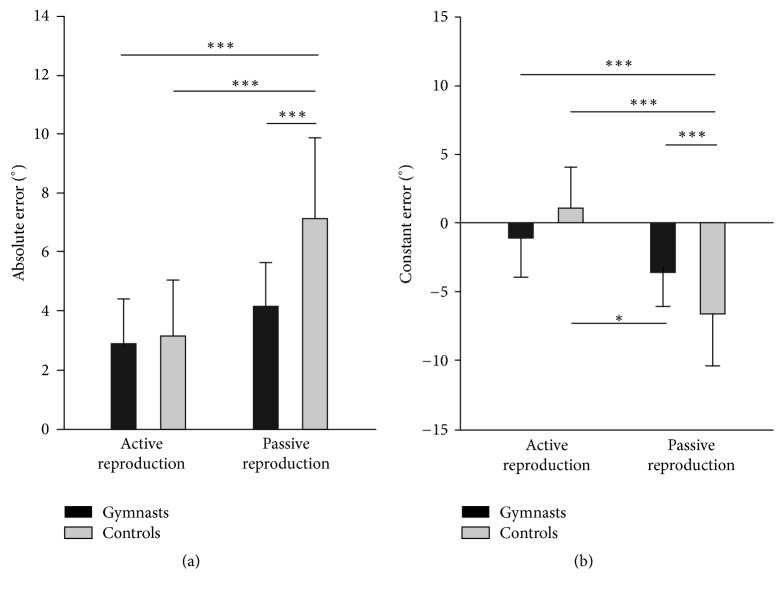
Joint position sense performance in the elbow joint. Absolute error (a) and constant error (b) are shown as mean ± SD. Significant difference at ^*∗*^*p* < 0.05 and ^*∗∗∗*^*p* < 0.001.

ANOVA test results, effect sizes, and post hoc test results for JPS are shown in Table 2. There was a significant main group effect on JPS performance. The gymnasts group had a 31% lower absolute error than the control group. The main mode effect was also significant with 96% greater absolute error in PR than in AR. The post hoc test of interaction of both effects showed that the above differences were due to higher absolute error in PR in the control group in comparison to the rest modes in either gymnasts or controls (Figure 1(a)). The analysis of the constant error showed similar results to the absolute error; however, the main group effect was insignificant. The main mode effect was significant, showing that participants during PR undershot the target angle by about 5.4 ± 3.5°, while during AR they slightly overshot it (0.1 ± 3.1°). The interaction of both effects showed that this undershot was mainly due to PR performance of the control group, which had significantly lower values of the constant error in comparison to AR and both reproduction modes in gymnasts (Figure 1(b)).

### 3.3. Force Sense

**Figure 2:**
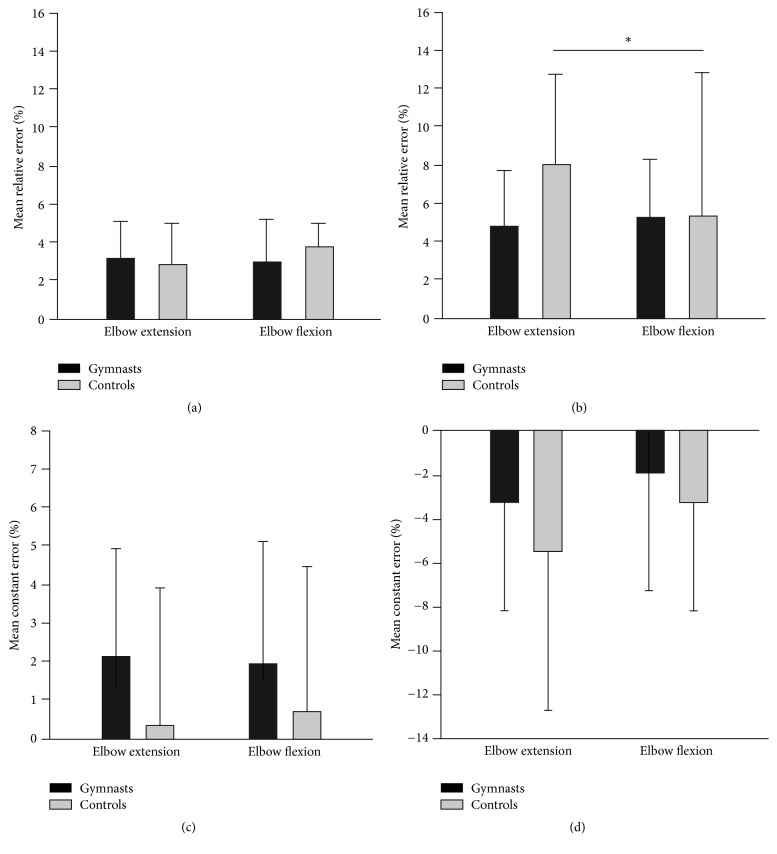
Force reproduction performance in the elbow joint. Mean relative and constant errors of 20% MVC (a, c) and 50% MVC (b, d) task are shown as mean ± SD. Significant difference at ^*∗*^*p* < 0.05.

ANOVA test results, effect sizes, and post hoc test results for FS are shown in Table 2. For 20% MVC, there was no significant main effect or interaction in the mean relative error (Figure 2(a)). The elbow extensors had a 10% higher range of relative error than the elbow flexors. For 50% MVC, there was a significant muscle effect as well as its interaction with the group effect in the mean relative error. The extensors had a 36% higher mean relative error in comparison to flexors. The post hoc test of interaction showed that these differences were due to 61% higher mean relative error values of elbow extensors in controls in comparison to their elbow flexors (Figure 2(b)). No effects were seen in the mean constant error. The only visible effect in the range of relative error was the muscle effect. In the extensor muscles, there was a 14% higher range of the relative error in comparison to flexor muscles. Gymnasts showed 10% higher results than controls, although the difference was insignificant.

### 3.4. Correlation Analysis

Pearson's correlation test results are shown in Table 3. Firstly, the relationship between JPS and FS performance was analyzed. A significant correlation was found between AR absolute error and the range of relative error for the 20% MVC flexion task (*r* = 0.48, *p* < 0.05) in the control group. For the same task, there was also a significant correlation between the AR absolute error and the FR mean constant error (*r* = 0.50, *p* < 0.05). No linear correlation was found with PR. In the gymnast group, there was also no correlation found for either AR or PR regarding the absolute error. On the other hand, gymnasts' AR constant error was significantly correlated with the mean constant and relative errors for 20% (*r* = −0.68, *p* < 0.05) and 50% (*r* = 0.49, *p* < 0.05) MVC extension tasks, respectively. Gymnasts' AR constant error was also correlated with the range of the relative error for 20% and 50% MVC extension tasks (*r* = −0.57, *p* < 0.05 and *r* = −0.62, *p* < 0.05, resp.). The outcome for gymnasts' PR constant error was similar, although instead of extension tasks it was correlated with the mean constant error in 20% (*r* = −0.49, *p* < 0.05) and the mean relative error in 50% (*r* = 0.56, *p* < 0.05) MVC flexion tasks. The PR constant error was also correlated with the range of the relative error for 20% MVC flexion task (*r* = −0.54, *p* < 0.05).

Secondly, the relationship between proprioception performance (FS, JPS) and MS was analyzed. For the 20% MVC flexion task, a significant correlation was found between mean relative error and absolute peak torque of elbow extension (*r* = −0.47, *p* < 0.05) in the control group. In the gymnast group, a significant correlation was found between ALM-normalized peak torque at the elbow extensors and both the mean relative and constant errors (*r* = −0.55–0.58, *p* < 0.05). For the 50% MVC flexion task, no significant correlations were found in either group. For the 20% MVC extension task, a linear significant correlation was found between controls' mean relative error and absolute peak torque in elbow flexion (*r* = −0.42, *p* < 0.05). No significant correlations were found in the gymnast group. Considering JPS, only in gymnasts was the AR constant error significantly correlated with ALM-normalized peak torque at the elbow flexors (*p* = 0.52, *p* < 0.05). No significant correlations were found between PR and MS variables.

## 4. Discussion

This study had two purposes. The first one was to evaluate the relationship between of MS, JPS, and FS, whereas the other one was to evaluate the impact of long-term gymnastic training on JPS and FS performance and the relationship between them. In the control group, a relationship between JPS and FS performance was seen only during the AR task. While the linear relationship between the accuracy of JPS and FR performance was insignificant, the AR higher absolute error was associated with overestimating the target force during 20% MVC flexion task. The absolute error of JPS during the AR task was also positively correlated with the range of the relative error during the same task, suggesting a possible common regulation mechanism with the steadiness of FR rather than with the accuracy of FS. One possible mechanism may be the sense of effort [34] which plays a role in the FS [9] but not in the PR task when the limb is supported [15]. However, in both modes, the reference position was set passively and therefore no additional effort cues (due to gravity force) should be transferred into the reproduction. Furthermore, the control group had lower absolute errors in AR in comparison to PR, which may suggest that muscle thixotropy was affecting results rather than additional effort cues [35]. This is consistent with the view that the muscle spindles are the main proprioceptors involved in JPS, as well as the view that effort has no effect on elbow JPS [36, 37]. Contrary to JPS, Scotland et al. [9] found that proprioceptors played little to no role during unilateral remembered FR tasks. Previous research has also found a relationship between force steadiness and postural control during quiet standing [38, 39], suggesting that the proprioceptive system has a common mechanism with force steadiness when muscles are being actively contracted. Interestingly, while gymnasts showed higher performance in PR than controls, there was no correlation between force steadiness and JPS accuracy in terms of the absolute error. This suggests that long-term gymnastic training has no impact on this relationship. However, the increased force steadiness was associated with overshooting the target angle in AR of JPS. On the other hand, gymnasts did not differ from controls during the AR task but did show a tendency towards a higher range of relative error during 20% MVC tasks. Therefore, changes in controlling the steadiness of FR could be responsible for group differences in the relationship between JPS and the steadiness of FR. It was expected that gymnasts would exhibit a substantially lower range of relative error (higher force steadiness), not the opposite, as it was shown that strength training is able to increase the force steadiness [40]. Nevertheless, it should be pointed out that, despite slightly worse steadiness, gymnasts had better accuracy in 50% MVC extension task. To summarize, despite intergroup differences in JPS and FS, these results support previous research demonstrating no linear correlation between accuracy of JPS and FS [19, 41].

The FR task in the study used MVC to set the target level of torque. It was therefore expected that FR performance would be correlated with agonist MS. However, a negative correlation was found between FR performance and peak torque of the antagonist muscle group. That is, the stronger the elbow extensors were, the lower the relative error during flexion task was, and vice versa. It is possible that the cocontraction of the antagonist muscle group contributes to joint stabilization allowing for greater FR accuracy [42, 43]. A lack of correlation between FR and agonist MS is consistent with previous research which found no significant correlation between agonist MS and FS in the knee joint of untrained adults [44]. In case of gymnasts, the relationship between antagonist MS and FS accuracy was seen only during 20% MVC flexion and involved ALM-normalized peak torque of extensors instead of absolute values. Normalization of peak torque as well as the muscle coactivation gives insight into neuromuscular coordination in muscle groups acting at particular joints [45–47]. It was expected that, due to long-term training, gymnasts would exhibit better neuromuscular coordination than untrained controls [26, 48]. In the study, gymnasts had higher normalized elbow flexion strength than controls. In addition, it was previously shown that gymnasts have lower coactivation of the antagonist muscles during the elbow flexion task [26]. These results suggest that gymnastic training decreases the torque reducing effect of extensor cocontraction during flexion tasks. This may explain the observed positive correlation of overshooting the target angle in AR of JPS with elbow flexors MS and also the previously discussed higher range of absolute error as the cocontraction of reciprocal muscle increased the steadiness [49].

Previous studies have found that physical activity and strength training can improve proprioceptive performance following injury [50]. However, Ashton-Miller et al. [25] noted that most of these studies did not evaluate the single elements of proprioception like JPS or kinesthetic threshold and focused instead on more complex postural control during balance tests. In the current study, it was shown that gymnasts had similar performance in both AR and PR, while the control group was twice as accurate during AR than during PR. The lower accuracy of the control group during PR was due to the fact that most of them underestimate the target angle. This suggests that long-term training allowed the gymnasts to overcome the thixotropic effect present during the PR task. One of the mechanisms responsible for that could be increased muscle spindle sensitivity and*γ*-afferent activation in the gymnast group [23]. This is supported by previous research which found that dancers demonstrate greater JPS performance at lower (1.5–2.5 times) [51] and upper (up to 3 times) limbs [52, 53] compared with untrained people. Also, athletes of other sports not associated with aesthetics and the control of body segments in space were shown to have 2.7 times better active JPS performance [54]. Furthermore, Pánics et al. [24] showed that proprioceptive training resulted in up to 2.5 times greater JPS performance among handball players. This suggests that JPS is trainable even in healthy subjects.

This study also investigated the difference in FR between the elbow flexors and extensors. Although no difference in FS accuracy was found during the 20% MVC task, a lower range of relative error in force steadiness was found in the flexor muscles. The opposite was found during the 50% MVC task with greater accuracy found in the flexor group. Both results of FS accuracy and force steadiness could be explained by the role and dominant muscle fibre type of each muscle group [55, 56]. The elbow flexors are meant for quick and precise movements while the extensors have a tonic and antigravity role. The lower accuracy of the extensors during the 50% MVC task was seen in the control group while in gymnasts there was no difference in FS accuracy between muscles. This outcome supports the theory that that long-term gymnastic training improves FS at the extensors during higher-load tasks but not during low-load tasks. Similarly, Smith et al. [57] showed that six weeks of strength training has no impact on the accuracy of FS at the ankle joint during 20% and 30% MVC tasks. This is probably the first time the muscle dependent effect of long-term training on the FS was shown. An interesting relationship was found between JPS and FR of antagonistic muscles in gymnasts. The overshoot performance in FR 20% MVC extension task was associated with underestimating the target angle in AR, while the same direction of bias during FR in flexion task gave the same result but in PR. This could also suggest that gymnastic training had a differentiating impact on the proprioception system in antagonistic muscles, but the mechanisms of this outcome, central, peripheral, or both, need future research.

*Limitations*. One of the study limitations was that the JPS measurements included only one target angle (90°) not based on the participants' total range of movement. While usage of additional target angles could increase the reliability of outcome, it was decided to preserve a similar elbow joint angle condition for the MS, JPS, and FR assessment. Moreover, such a target angle avoids additional cues from the soft tissue stretch, and apposition at the ends of range influences the accuracy of JPS [58]. The second limitation is that the study's design was unable to account for the role of Golgi tendon organs in the measured proprioception performance. While the receptors' afferent information contributes to FS, many authors show that their role might be less present due to the central mechanism of proprioception.

## 5. Conclusions

While there is no evidence for a linear relationship between the accuracy of FS and JPS, force steadiness and JPS may use common mechanisms. Long-term gymnastic training appears to result in greater JPS accuracy performance and to reduce the difference in FS between reciprocal muscle groups at the elbow joint by increasing accuracy at the extensor muscles.

## Figures and Tables

**Table 1:** Anthropometric and muscle strength characteristics of participants.

	Gymnasts (*n* = 17)	Nontrained (*n* = 24)
	Mean ± SD	Mean ± SD
Age (years)	20.54 ± 3.51	19.84 ± 0.93
Body mass (kg)	68.26 ± 7.02	73.23 ± 8.49
Height (cm)	170.32 ± 4.16	177.96 ± 4.90
Body mass index (kg·m^−2^)	23.48 ± 1.59	23.14 ± 2.49
Body fat (%)	7.37 ± 4.27^*∗∗*^	11.65 ± 3.16
Arm lean mass (kg)	4.02 ± 0.57	3.77 ± 0.61
Elbow flexion peak torque		
Absolute (N)	66.21 ± 13.30^*∗∗∗*^	49.97 ± 12.11
Arm lean mass, normalized (N·kg^−1^)	16.52 ± 2.43^*∗∗∗*^	13.36 ± 2.87
Elbow extension peak torque		
Absolute (N)	70.15 ± 15.26	64.70 ± 14.31
Arm lean mass, normalized (N·kg^−1^)	17.42 ± 2.54	17.16 ± 2.97

Significant difference between groups at ^*∗*^*p* < 0.05, ^*∗∗*^*p* < 0.01, and ^*∗∗∗*^*p* < 0.001.

**Table 2:** Results of the two-way ANOVA with repeated measures in elbow JPS and FR performance.

Task	Indicator	Effect	*F*	*p*	Effect size (*η*^2^)	Post hoc
JPS	Absolute error	Group × mode	8.47	<0.01^*∗∗*^	0.18	PR: C > AR: C, G
		Group	13.26	<0.01^*∗∗*^	0.25	G < C
		Mode	30.37	<0.01^*∗∗∗*^	0.43	PR > AR
	Constant error	Group × mode	17.52	<0.01^*∗∗*^	0.31	C: PR < G: PR, AR; PR: C, G < AR: C
		Group	0.40	0.53	0.01	
		Mode	66.59	<0.01^*∗∗∗*^	0.63	PR < AR

FR 20% MVC	Mean relative error	Group × muscle	0.57	0.45	0.02	
		Group	0.21	0.65	<0.01	
		Muscle	0.57	0.45	0.02	
	Mean constant error	Group × muscle	0.24	0.63	<0.01	
		Group	2.96	0.09	0.07	
		Muscle	0.01	0.91	<0.01	
	Range of relative error	Group × muscle	0.06	0.81	<0.01	
		Group	3.84	0.06	0.09	
		Muscle	7.38	<0.01^*∗∗*^	0.15	Extension > flexion

FR 50% MVC	Mean relative error	Group × muscle	4.86	0.03^*∗*^	0.11	C: Extension > flexion
		Group	1.91	0.17	0.05	
		Muscle	4.53	0.04^*∗*^	0.10	Extension > flexion
	Mean constant error	Group × muscle	2.24	0.14	0.05	
		Group	2.72	0.11	0.07	
		Muscle	1.21	0.27	0.03	
	Range of relative error	Group × muscle	1.20	0.28	0.03	
		Group	0.33	0.57	<0.01	
		Muscle	0.18	0.68	<0.01	

JPS: joint position sense; FR: force reproduction; MVC: maximal voluntary contraction; PR: passive reproduction; AR: active reproduction; C: control group; G: gymnasts. Significant effect or interaction at ^*∗*^*p* < 0.05, ^*∗∗*^*p* < 0.01, and ^*∗∗∗*^*p* < 0.001.

**Table 3:** Pearson's correlation analysis of the muscle strength and proprioception performance.

	Joint position sense				Muscle strength			
	AR		PR		PT flexion		PT extension	
	AE	CE	AE	CE	AV	ALM	AV	ALM
Joint position sense AR								
AE	/	/	/	/	ns	ns	ns	ns
CE	/	/	/	/	ns	0.52 (G)	ns	ns
Joint position sense PR								
AE	/	/	/	/	ns	ns	ns	ns
CE	/	/	/	/	ns	ns	ns	ns
FR 20% MVC flexion								
Mean RE	ns	ns	ns	ns	ns	ns	−0.47 (C)	−0.55 (G)
Mean CE	0.50 (C)^*∗*^	ns	ns	−0.49 (G)	ns	ns	ns	−0.58 (G)
RE range	0.48 (C)	ns	ns	−0.54 (G)	ns	ns	ns	ns
FR 20% MVC extension								
Mean RE	ns	ns	ns	ns	−0.42 (C)	ns	ns	ns
Mean CE	ns	−0.68 (G)	ns	ns	ns	ns	ns	ns
RE range	ns	−0.57 (G)	ns	ns	ns	ns	ns	ns
FR 50% MVC flexion								
Mean RE	ns	0.49 (G)	ns	0.56 (G)	ns	ns	ns	ns
Mean CE	ns	ns	ns	ns	ns	ns	ns	ns
RE range	ns	ns	ns	ns	ns	ns	ns	ns
FR 50% MVC extension								
Mean RE	ns	ns	ns	ns	ns	ns	ns	ns
Mean CE	ns	ns	ns	ns	ns	ns	ns	ns
RE range	ns	−0.62 (G)	ns	ns	ns	ns	ns	ns

/ indicates the correlation between the same proprioceptive performances, which was not analyzed. AE: absolute error; ALM: arm lean mass normalized values; AR: active reproduction; AV: absolute values; CE: constant error; FR: force reproduction; MVC: maximal voluntary contraction; PR: passive reproduction; PT: peak torque; RE: relative error. ^*∗*^(G) and (C) indicate significant correlation in gymnasts and controls, respectively (*p* < 0.05), and “ns” indicates nonsignificant correlation.
